# Dual-Surface-Modified Triboelectric Nanogenerator with Polymer Microcone Array and Its Application to Impact Visual and Voice Warning

**DOI:** 10.3390/polym17111569

**Published:** 2025-06-05

**Authors:** Dong-Yi Lin, Chen-Kuei Chung

**Affiliations:** Department of Mechanical Engineering, National Cheng Kung University, Tainan 701, Taiwan

**Keywords:** triboelectric nanogenerators, dual-surface modify, cold imprint, polymer, self-power sensor, voice–visual warning

## Abstract

Poly(dimethylsiloxane) (PDMS) is a predominantly utilized negative triboelectric material in triboelectric nanogenerators (TENGs). Its surface topography and synergistic interaction with positive triboelectric materials significantly impact the performance of TENGs. Here, we propose a simple and cost-effective approach to promote the performance of a dual-surface-modified TENG using microwave-structured aluminum (MW-Al) together with microcone-structured polydimethylsiloxane (MC-PDMS). Laser-engraved molds were employed to cold-imprint the MC-Al and pattern the MC-PDMS. Subsequently, the impact of the heights of microcones generated under varying laser powers on the performance of TENGs was explored. The output performance of the MW-MC-TENG significantly increased with microcone heights from 0 to 228 μm. The MW-MC228-TENG, with the highest cone heights, can produce the best open-circuit voltage of 157 V and a short-circuit current of 78.5 µA, resulting in a more than 37% improvement compared to the TENG using flat polymer. Furthermore, the MW-MC228-TENG showed a power density of 16.4 W/m^2^, sufficient to power 198 LEDs. Finally, the proposed TENG was integrated as a sensor into an impact warning system. We triggered a voice–visual warning when the TENG impacted, proving its potential for intelligent home safety monitoring.

## 1. Introduction

With the continuous advancement of digital information technology and electronic sensing technologies, human society has entered the era of the Internet of Things (IoT) [1]. The IoT has permeated various aspects of daily life, including industrial manufacturing, transportation, environmental protection, smart homes, healthcare, and human sensing, enhancing convenience and progress in human society [2]. Although each IoT device consumes very little power, the cumulative power consumption of all IoT devices will be unimaginable, creating a substantial demand for mobile energy solutions [3]. Therefore, effective collection and utilization of renewable energy, such as marine [4], wind [5], solar [6], and geothermal energy [7], represent ideal choices for advancing the global economy and human civilization. In recent years, various green energy collectors based on thermoelectric/pyroelectric [8], electromagnetic [9], piezoelectric [10], triboelectric [3,8], and solar cell (SC) mechanisms [11,12] that can effectively collect energy from the surrounding environment have been widely studied and developed. In 2012, Wang et al. first reported the triboelectric nanogenerator (TENG), a device that converts mechanical energy from the environment into electrical energy through the coupling of contact electrification and electrostatic induction [13]. Since their introduction, TENGs have demonstrated significant potential across multiple domains, including the IoT [14], 5G [15], and intelligent systems [16]. Their broad applicability spans energy harvesting [17], self-powered sensing [18], battery-free devices [19], medication [20], and human–computer interaction [21]. These advantages stem from their low cost, lightweight design, material versatility, high power density, and high efficiency even at low operating frequencies [13,18]. In self-powered sensing applications, triboelectric nanogenerators (TENGs) have demonstrated significant utility across diverse domains, including self-powered lantern [12], signal receiver [15], personalized healthcare [16], electric vehicles [16], artificial intelligence [18], wireless sensor systems [19], and human–computer interaction [21].

The electrical performance of TENGs primarily depends on the structural design, material selection, surface modification, and friction characteristics of materials. In conventional TENG fabrication, material pairs exhibiting distinct and opposing triboelectric polarities are typically selected as contact layers to optimize charge generation [22]. The selection of optimal triboelectric material pairs constitutes a fundamental consideration in TENG design, as it directly governs charge transfer efficiency and overall device performance. Surface roughness, thickness, contact area, stiffness, and charge affinity are essential material parameters [23,24]. Among existing optimization strategies, the electrical output of triboelectric nanogenerators (TENGs) can be significantly enhanced by controllably expanding the effective contact area through double-sided micro/nano-patterning of triboelectric surfaces [25,26]. In recent years, numerous double-sided micro/nano-patterned architectures have been developed to enhance TENG performance (Table 1), including microporous-scale morphology [25], microgroove-microgroove [26], nanofiber–nanofiber [27], sandpaper morphology-nanofiber [28], nanoporous-nanoporous [29], nanopillar–nanopillar [30], or microwave-microcone [31]. However, these nano/micro-structures are fabricated mainly by methods such as etching [25,26], sputtering, lithography [26], electrospinning [27], ecoflex casting [28], chemical functionalization [29], and electrodeposition [30]. These techniques are time-consuming and costly, limiting the continued development and broader application of TENGs. In our previous research [31], we proposed a simple and low-cost method for preparing double-sided patterned TENGs, which demonstrated excellent electrical output performance. Nevertheless, several limitations were identified, suggesting opportunities for further enhancement.

In this article, we propose a TENG device with enhanced electrical performance featuring double-sided patterned friction pairs. The friction pairs consist of a patterned aluminum electrode featuring periodic microwave (MW) array structures and a polydimethylsiloxane (PDMS) triboelectric layer with complementary microcone (MC) arrays. The aluminum tribo-layer was patterned using a cold imprinting method. The PDMS tribo-layer was fabricated via an integrated CO_2_ laser ablation [32,33,34,35] and rapid prototyping casting process, ensuring an excellent match between the MW and MC arrays. During the cold imprinting process of the aluminum film, a polymethylmethacrylate (PMMA) mold with micro-hole arrays, also fabricated via CO_2_ laser ablation, was utilized as the compression mold. To facilitate comparison, we fabricated three kinds of low-cost, simple triboelectric PDMS materials with varying physical structures and surface topologies. The specific parameters of these materials are outlined in Table 2. These include (1) untreated PDMS film (flat-PDMS), (2) PDMS film with a 56 µm microcone height (MC56-PDMS), and (3) PDMS film with a 228 µm microcone height (MC228-PDMS). The triboelectric performance of these materials was evaluated by fabricating double-sided patterned TENGs in vertical contact–separation mode, using Al as the positive material, PMMA as the substrate, and PDMS as the negative material.

The electrical performance parameters were measured for three TENGs as shown in Table 2, revealing the following trend: MW-flat-TENG < MW-MC56-TENG < MW-MC228-TENG. Specifically, the MW-MC228-TENG demonstrated superior performance, with *V_oc_*, *I_sc_*, and *J_sc_* values of 157 V, 78.5 µA, and 3.14 µA/cm^2^, respectively. These values demonstrate significant performance improvements of 37.7%, 38.9%, and 38.9%, respectively, relative to the MW-flat-TENG. These improvements are attributed to the synergistic combination of MW-Al porosity and the increased surface roughness of MC228-PDMS. Consequently, MC228-PDMS was identified as the optimal negative material for MW-MC-TENG. Furthermore, the MW-MC228-TENG exhibited practical utility by charging a 0.1 µF capacitor to 2 V within 1 s and powering 198 green LEDs connected in series. Furthermore, the MW-MC228-TENG was effectively implemented as a self-powered impact alarm sensor. This application successfully achieved collision detection for potential hazards affecting visually impaired individuals or children, while concurrently transmitting real-time signals to both a human–machine interface (HMI) system [36] and an LED array or immediate visual alerts. This functionality enables real-time warnings, demonstrating its potential for integration into IoT applications.

## 2. Materials and Methods

### 2.1. Fabrication of Tribo-Pairs

**Figure 1:**
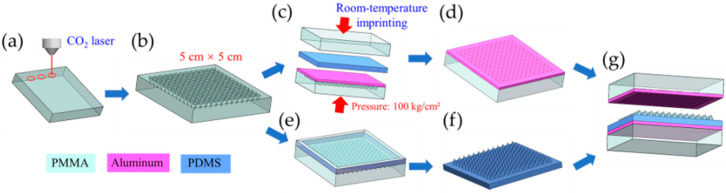
Fabrication process and assembly schematic of MW-MC-TENG: (**a**) CO_2_ laser ablation patterning of PMMA substrate. (**b**) Resultant PMMA master mold microstructure. (**c**) Pressure-assisted embedding of conductive aluminum tape into PMMA mold using planar PMMA plate and PDMS elastomer interlayer. (**d**) Formation of positive triboelectric layer adhered to PMMA mold after removal of temporary PMMA plate and PDMS spacer. (**e**) Casting of PDMS prepolymer solution into secondary PMMA mold. (**f**) Cured negative triboelectric layer after thermal treatment and demolding. (**g**) Schematic of fully assembled Al/PDMS-based TENG device.

**Fabrication of MW**-**Al.** Consistent with our previous research [31], the MW-Al electrode was fabricated using the following procedure: A PMMA master mold featuring a microporous array was created via CO_2_ laser ablation (power = 0.06 W), as illustrated in Figure 1a,b. The array design was developed using CorelDraw X7 software, and the CO_2_ laser processing parameters were controlled using a laser system (VL-200, General Laser System, Scottsdale, AZ, USA). A 60 µm thick conductive Al tape was adhered to the PMMA surface within a 5 cm × 5 cm area, completely covering the microporous array. A pre-cured, planar PDMS layer was subsequently placed between a planar PMMA and the Al tape, forming a sandwich-like structure. Subsequently, a pressure of 100 kg/cm^2^ was exerted on the assembly using a hydraulic press, as depicted in Figure 1c. The resultant MW-Al composite structure on the PMMA substrate functioned simultaneously as the upper triboelectric layer and working electrode, as illustrated in Figure 1d.

**Preparation of MC-PDMS.** Similar to our previous research [31], the PMMA master molds for MC56 and MC228 were fabricated using CO_2_ laser ablation (Figure 1a). The laser powers were set to 0.06 W and 0.6 W, respectively. The scanning speed was maintained at 11.4 mm/s, and the pulse per inch (PPI) was set to 110 µm, as outlined in Table 2. The PDMS prepolymer was thoroughly mixed with the curing agent at a 10:1 mass ratio and carefully cast into the laser-ablated PMMA master molds (Figure 1e). The prepared mixture was degassed under vacuum for 10 min to eliminate air bubbles and ensure complete cavity filling. The PDMS was subsequently cured in an oven at 85 °C for 1 h. The cured PDMS film was then carefully demolded from the PMMA template, sectioned into 5 cm × 5 cm square specimens, and meticulously trimmed to remove peripheral irregularities, as demonstrated in Figure 1f. The fabricated PDMS films—including flat-PDMS, MC56-PDMS, and MC228-PDMS variants—were produced with identical dimensions and consistent thickness.

**Assembly of MW-MC-TENG.** Figure 1g illustrates the component materials for MW-MC-TENG assembly: (1) two PMMA substrate plates, (2) one microstructured MC-PDMS triboelectric layer, (3) one porous MW-Al foil electrode, and (4) one flat aluminum counter electrode. During the assembly procedure, the MC-PDMS film was adhered to the flat Al plate to act as the top electrode, whereas the MW-Al foil was imprinted onto and attached to the PMMA mold to function as the bottom electrode, thereby finalizing the assembly of the MW-MC-TENG.

### 2.2. Performance Characterization of the MW-MC-TENG

**Figure 2:**
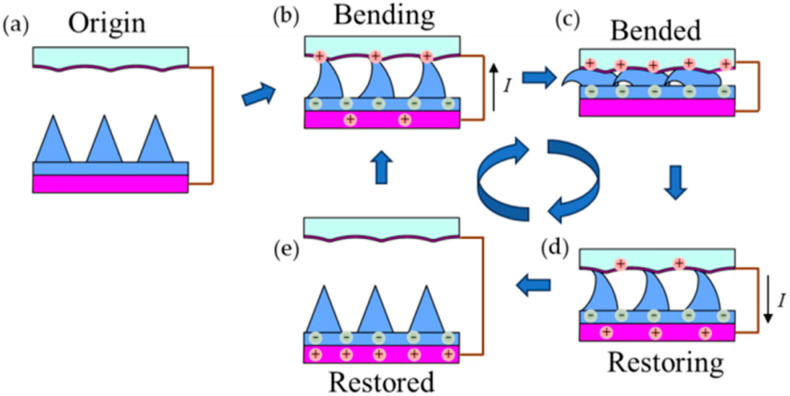
Operational mechanism and measurement configuration of the MW-MC-TENG: (**a**) Initial equilibrium state prior to contact. (**b**) Maximum charge transfer during complete interfacial contact, demonstrating triboelectric polarization. (**c**) Separation phase showing electrostatic induction effects. (**d**) Full elastic recovery of the MC-PDMS microstructure to its original geometry. (**e**) Compression cycle illustrating contact electrification renewal.

The output performance of the MW-MC-TENG was characterized using a vertical contact–separation test system. A pneumatic cylinder (FESTO, model: DSNU-20-100-PPV, Esslingen, Germany) with controllable actuation provided consistent periodic contact at a controlled frequency (0–10 Hz) and force (0–50 N), while the generated electrical signals were simultaneously recorded. Figure 2 presents the operational schematic and working principle of the MW-MC-TENG, illustrating both the measurement configuration and fundamental charge transfer mechanisms during contact–separation cycles. The MW-Al electrode and MC-PDMS triboelectric layer were mounted on opposing components, with the MW-Al adhered to the actuator shaft and the MC-PDMS affixed to the stationary stage. The TENG electrodes comprised (1) a planar aluminum mounted on the stationary stage and (2) the porous MW-Al composite layer bonded to the PMMA substrate. The pneumatic testing platform was operated at 196 kPa (2 kg/cm^2^) input pressure, maintaining a 20 mm separation distance between the test specimen and MW-Al electrode during cyclic contact–separation testing. The aluminum electrode and the test piece were connected via copper wires to a transient waveform recorder to measure the open-circuit voltage during testing. The circuit was connected in parallel with a high-resistance load to measure the short-circuit current. All the electrical output performance tests are operated at 22~25 °C, 60~65% humidity. Finally, the *V_oc_* and *I_sc_* of the TENGs were characterized by an oscilloscope (HIOKI Memory HiCorder MR8870-20, Nagano, Japan).

### 2.3. Characterization of Materials

The microstructure of MW-Al and MC-PDMS was characterized by optical microscopy (OM; Olympus BX 51M, Tokyo, Japan). The surface morphology and microstructural topography of the MC-PDMS films were characterized using field emission scanning electron microscopy (FE-SEM; Hitachi SU-4800, 5 kV accelerating voltage) and atomic force microscopy (AFM; Bruker Dimension Icon, ScanAsyst mode in air). The Kelvin probe force microscopy (KPFM) employed in this study was integrated with the AFM controller.

## 3. Results

### 3.1. The Micromorphology Structure of the Samples

**Figure 3:**
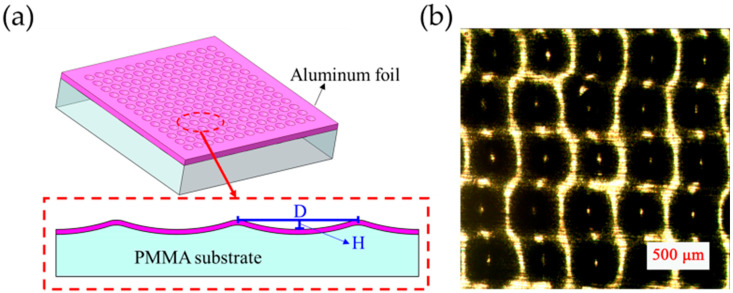
(**a**) Sectional schematic diagram of MW-Al friction foil with microwave height of ~18 µm; (**b**) OM image of MW-Al foil.

Figure 3 characterizes the microstructural morphology of the porous MW-Al foil, showing: (a) a cross-sectional view revealing wavy topography with wave height (H) ≈ 18 μm and (b) a planar view demonstrating a periodic grid pattern with a uniform pore array (average interpore spacing D ≈ 250 μm). The three-dimensional architecture combines both waviness and through-pores, enhancing surface area for triboelectric applications.

**Figure 4:**
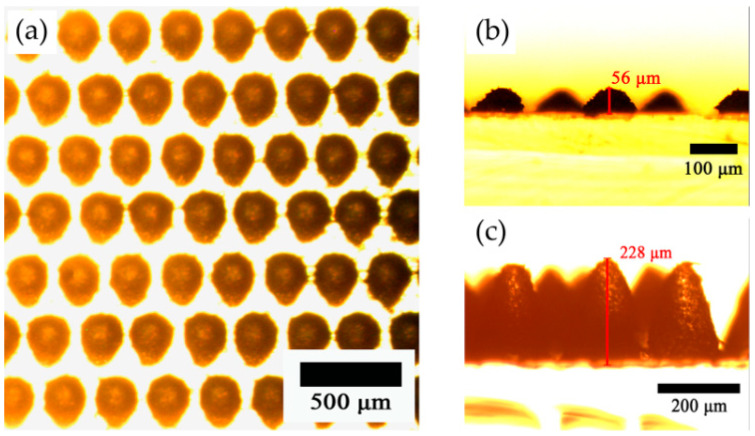
The surface morphology of the microcone PDMS structure. (**a**) OM image from the top view. OM image of (**b**) MC56-PDMS and (**c**) MC228-PDMS from the front view.

Figure 4 presents the surface morphology of demolded MC-PDMS replicas, demonstrating (a) uniform microcone arrays with consistent ~250 μm periodicity across all samples; optical microscopy reveals distinct geometric profiles for (b) MC56-PDMS (height ≈ 56 μm, base diameter ≈ 118.6 μm) and (c) MC228-PDMS (height ≈ 228 μm, base diameter ≈ 174.3 μm), corresponding to laser ablation powers of 0.06 W and 0.6 W, respectively. All replicas maintained a uniform PDMS substrate thickness of ~700 μm, as quantified in Table 3.

**Figure 5:**
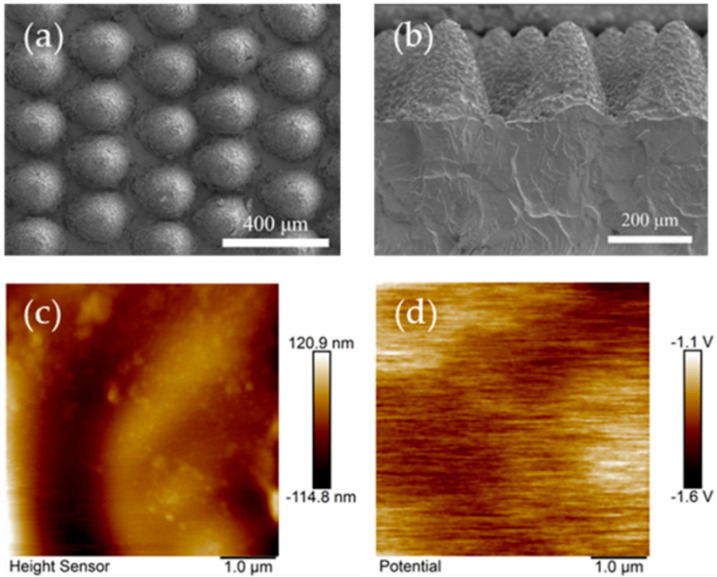
SEM images of the MC228-PDMS: (**a**) Top view and (**b**) sectional view. (**c**) AFM topographic image and (**d**) KPFM potential image of the MC228-PDMS.

Given the enhanced contact area and bending deformation capability of MC228-PDMS, we performed comprehensive surface characterization. Scanning electron microscopy (SEM) revealed (a) top-view and (b) cross-sectional morphologies, showing laser-induced burrs distributed periodically across microcone surfaces (Figure 5a,b). These nanostructured features significantly increase surface roughness and effective contact area. Quantitative atomic force microscopy (AFM) analysis (Figure 5c) yielded an average roughness (R_a_) of 118.3 nm and root-mean-square roughness (R_q_) of 168.7 nm. Kelvin probe force microscopy (KPFM) measurements (Figure 5d) demonstrated an elevated surface potential of 20.8 mV, confirming that the laser-generated surface asperities enhance both topographic and electronic surface properties critical for triboelectric performance.

### 3.2. Working Principle of TENG

The MW-MC-TENG operates through the synergistic coupling of contact electrification and electrostatic induction mechanisms. The triboelectric material pairing (MC-PDMS/MW-Al) and electrode configuration synergistically govern the charge generation and transfer processes, as schematically depicted in Figure 2. Firstly, Figure 2a illustrates the initial equilibrium state of the MW-MC-TENG, in which no charge generation takes place. Figure 2b shows the MC-PDMS in a bent state during the pressing stroke, triggering dipole triboelectric charging. At this stage, electrostatic induction drives charge redistribution, generating a transient electron flux from the upper electrode to the bottom electrode through the external circuit. Figure 2c illustrates the elastomeric microcones in their deformed state during tribological engagement, showing full conformal contact between friction pairs and asymmetric bending of microcone structures along the sliding direction under applied normal pressure. At this stage, electrostatic equilibrium is achieved as equivalent triboelectric charge densities develop on both contact surfaces. Consistent with their relative positions in the triboelectric series, electron transfer during contact electrification results in positive charges on the MW-Al surface and negative charges on the MC-PDMS interface. Figure 2d illustrates the elastic recovery of MC-PDMS microstructures upon pressure release, initiating charge layer separation and the subsequent electrostatic induction phase. To equilibrate the established potential difference, conduction electrons migrate from the bottom electrode to the top electrode through the external circuit, producing a reverse current pulse. Figure 2e shows the complete recovery of the MC-PDMS to its original shape, with the two frictional layers reaching their maximum separation distance and the electron flow stopping, thus achieving electrostatic equilibrium. Cyclic contact and separation motion between the pairs drives continuous triboelectric charge transfer and dynamic interfacial charge redistribution.

### 3.3. Output Performance of the TENG

**Figure 6:**
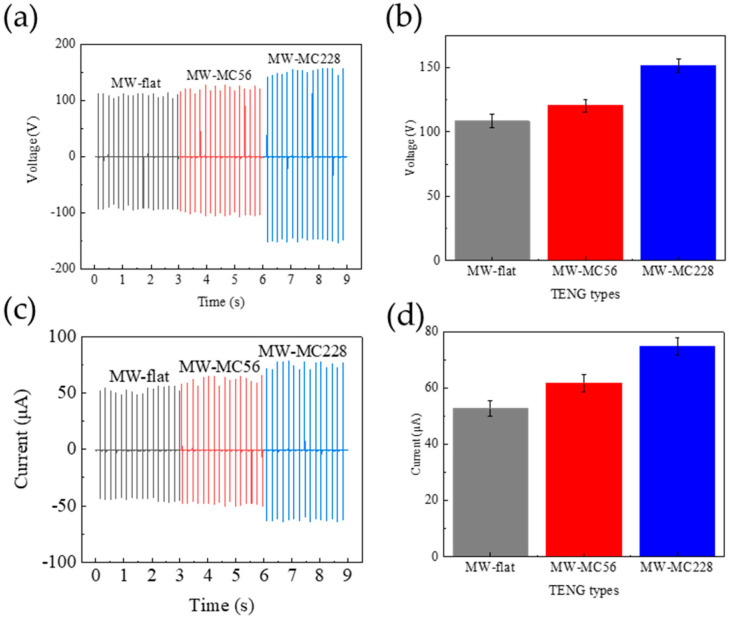
Comparative electrical output characteristics of MW-MC-TENG devices: (**a**,**b**) *V_oc_* and (**c**,**d**) *I_sc_* measurements for (i) MW-flat-TENG (control), (ii) MW-MC56-TENG, and (iii) MW-MC228-TENG configurations.

To systematically evaluate microstructure effects on TENG performance, we fabricated three distinct microstructured PDMS variants (along with an untreated control) and paired them with MW-Al electrodes to construct comparative device architectures. The pneumatic testing system maintained fixed contact–separation parameters (frequency: 6 Hz, normal force: 30 N) throughout the experiments. The *V_oc_*, *I_sc_*, and *J_sc_* of the TENGs are shown in Table 2 and Figure 6. The comparison results in Figure 6a,c indicate that the order of output performance of the three TENGs is as follows: MW-flat-TENG (114 V, 56.5 μA, 2.26 μA/cm^2^) < MW-MC56-TENG (127 V, 66 μA, 2.64 μA/cm^2^) < MW-MC228-TENG (157 V, 78.5 μA, 3.14 μA/cm^2^). The TENG output performance exhibited a strong positive correlation with microcone height, demonstrating statistically significant enhancement in both voltage (Figure 6b) and current (Figure 6d) outputs across the tested geometrical gradient. The electrical output performance of this MW-MC228-TENG is also higher than that (141 V, 71.5 µA, and 2.86 µA/cm^2^) of the TENG with a microcone height of 180 μm (MW-MC180-TENG) that we previously studied [31]. The surface microstructural topography of triboelectric interfaces significantly influences MW-MC-TENG output characteristics. The *V_oc_*, *I_sc_*, and *J_sc_* of the MW-MC228-TENG reach their peak values mainly due to the higher height and diameter of the PDMS microcones compared to the other samples. This results in greater bending, deformation, and friction, further enhancing the effective contact area when pressed. As a result, the MC228-PDMS was chosen as the friction layer and charged electrode for the MW-MC-TENG.

**Figure 7:**
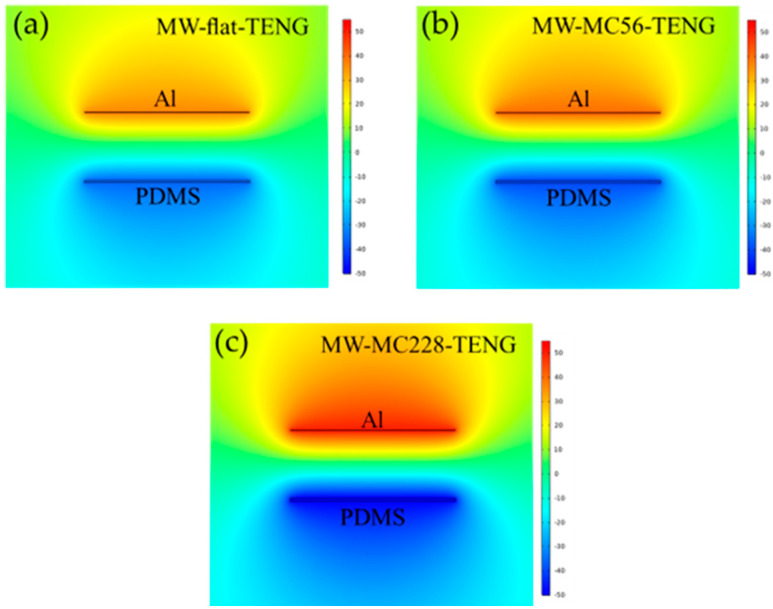
COMSOL Multiphysics^®^ simulations of contact–separation mode dynamics for three TENG architectures: (**a**) planar reference (MW-flat-TENG), (**b**) intermediate microstructure (MW-MC56-TENG), and (**c**) optimized microstructure (MW-MC228-TENG).

To systematically analyze microstructure-dependent surface potential characteristics, we conducted finite element simulations (COMSOL Multiphysics^®^ v5.6) comparing the three TENG architectures, with results presented in Figure 7. The same frictional layer size (5 cm × 5 cm) and gap distance (20 mm) were set in COMSOL for all simulations. The calculated triboelectric charge densities for the three TENG configurations were quantitatively determined as 48.4 nC/cm^2^ (flat), 53.5 nC/cm^2^ (MC56), and 67.0 nC/cm^2^ (MC228), reflecting the enhanced charge transfer efficiency enabled by microstructured interfaces. The circular air environment models are used with an 80 mm radius boundary and 10 mm circular layer thickness. The relative dielectric constants of air, MW-Al, and MC-PDMS are 1, 1, and 2.75, respectively. The Young’s moduli of MW-Al, and MC-PDMS are 7 × 10^4^ MPa, and 750 kPa, respectively. Ultra-fine meshes, including 560 quadrilateral elements and 13,050 triangular elements with sizes ranging from 0.012 to 3.2, are used to mesh the circular air layer and the remaining material, respectively. The simulated output voltages exhibited progressive enhancement with microstructure optimization: (i) MW-flat-TENG: 75 V (Figure 7a), (ii) MW-MC56-TENG: 80 V (Figure 7b), and (iii) MW-MC228-TENG: 99 V (Figure 7c). This 32% voltage improvement correlates strongly with increasing surface roughness, confirming the critical role of microstructural topography in enhancing triboelectric charge generation.

**Figure 8:**
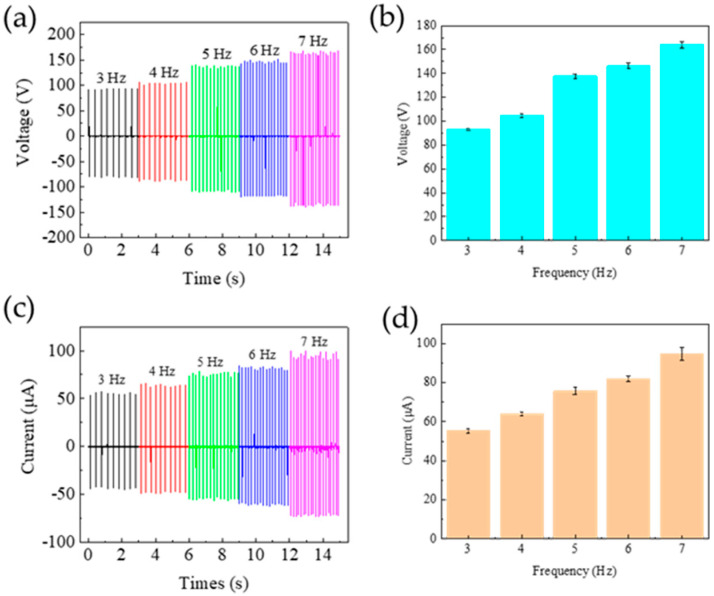
Frequency-dependent output characteristics of MW-MC228-TENG: (**a**,**b**) open-circuit voltage and (**c**,**d**) short-circuit current spectra across 3-7 Hz operational frequencies.

The frequency-dependent output characteristics of the MW-MC228-TENG were systematically characterized across 3–7 Hz under controlled conditions (fixed 20 mm separation distance), as quantitatively analyzed in Figure 8. The *V_oc_* and *I_sc_* gradually increase with the stamping frequency. They, respectively, reach maximum values of 169 V and 79 μA under maximum excitation frequency. The increasing contact–separation frequency induces a proportional reduction in interfacial contact duration. This reduction leads to a decrease in the quantity of charge on the electrode plate, thereby facilitating an increase in both the *V_oc_* and the *I_sc_*.

### 3.4. Improvement Mechanism of the TENG

**Figure 9:**
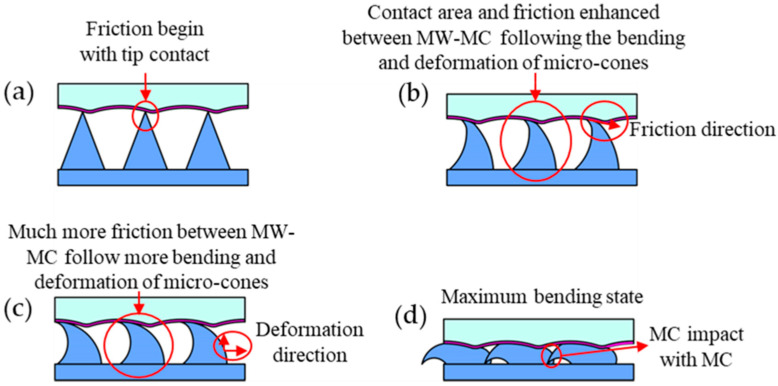
Mechanism of MW-MC-TENG performance enhancement during microcone compression deformation. (**a**) Original state. (**b**) Initial contact with small areas. (**c**) Expansion of contact area and frictional interaction with progressive deformation. (**d**) Contact enhancement under maximum deformation.

Figure 9 elucidates the multi-physics enhancement principle of the MW-MC-TENG using a 2D schematic model, demonstrating the deformation and restoration processes of the microcones (MCs) during a cyclic contact–separation process. The schematic diagram delineates each phase sequentially. In Figure 9a, the initial contact state between the two tribo pairs is shown, where MW-Al and MC-PDMS remain in a non-interacted state, and tip contact and deformation are confined to the MCs’ apexes. Figure 9b depicts an increased contact and friction state, where bending and deformation of the microcones enlarge the contact area, initiating dipole triboelectric charging. Figure 9c illustrates further progression of contact and friction, with a substantial expansion in the contact area and greater bending deformation of the MCs. Finally, Figure 9d presents the full contact state, where the triboelectric charge distribution achieves maximum equilibrium between the two materials. At this stage, the MCs’ bending and deformation peak, maximizing the contact area and enhancing the MW-MC-TENG’s performance, yielding peak voltage and current outputs.

Figure 9 and Table 2 quantitatively demonstrate that the microcavity (MC) morphology and surface area dominantly influence the deformation behavior and frictional dynamics. Larger MCs possess greater volume and surface area, increasing the deformation and friction rate, assuming a consistent cone density across the three samples. This is the primary reason for the enhanced voltage and current of the MW-MC228-TENG compared to the other TENGs.

### 3.5. Power, Charging Capability, and Durability

**Figure 10:**
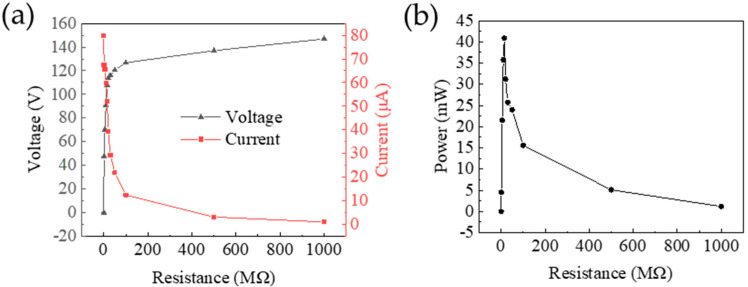
Electrical output characteristics of the MW-MC-TENG versus load resistance: (**a**) voltage and current responses; (**b**) power output.

To determine the peak output power of the TENG, external resistors (1 kΩ to 1 GΩ) were connected in series within the test circuit. The output voltage exhibits a rapid initial increase followed by a gradual approach to saturation as the resistance increases (Figure 10a). In contrast, the output current exhibits a gradual decrease. At a 15 MΩ load resistance, the MW-MC-TENG achieves peak performance with 107.9 V output voltage and 52.2 µA current, yielding a maximum power output of 40.9 mW (Figure 10b). This corresponds to an exceptional power density of 16.4 W/m^2^.

**Figure 11:**
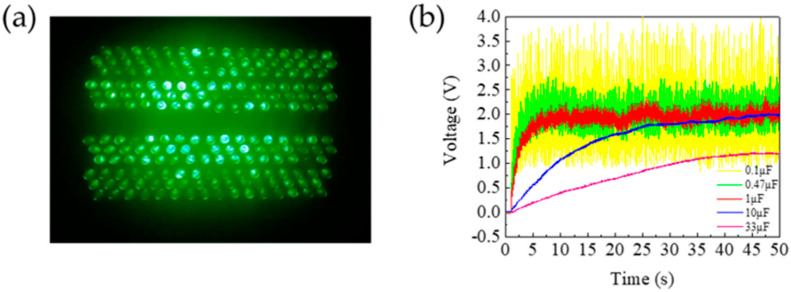
Energy harvesting applications of the MW-MC-TENG: (**a**) photograph of 198 series-connected LEDs powered by the device; (**b**) capacitive charging characteristics for 0.1–33 μF capacitors.

To assess the performance of the fabricated TENG as a power supply source for small electronic devices, we evaluated the MW-MC-TENG’s capability to illuminate commercial LEDs and charge capacitors. The alternating current output was converted to direct current using a bridge rectifier before connecting to external loads. As demonstrated in Figure 11a, the MW-MC-TENG successfully powered 198 series-connected green LEDs (1.9 V, 665 mW total power requirement). Figure 11b shows the charging characteristics for five capacitors ranging from 0.1 μF to 33 μF. The device achieved charging voltages of 2 V (0.1 μF and 0.47 μF), 1.9 V (1 μF and 10 μF), and 1.2 V (33 μF) within 1 s, 5 s, 8 s, 45 s, and 45 s, respectively. These results demonstrate that the MW-MC-TENG serves as an effective self-powered energy source for driving commercial LEDs or charging capacitors, offering distinct advantages over traditional batteries for powering small IoT devices or functioning as self-powered sensors in intelligent systems.

**Figure 12:**
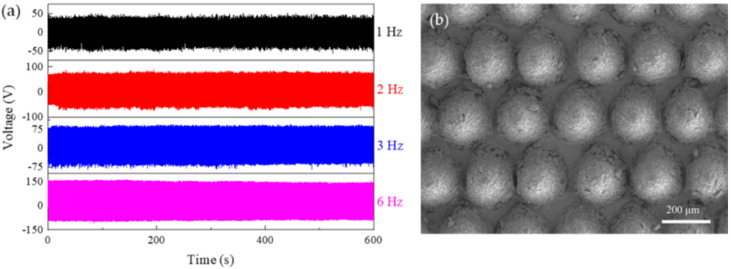
(**a**) Stability testing of MW-MC228-TENG at operating frequencies of 1 Hz, 2 Hz, 3 Hz, and 6 Hz. (**b**) The SEM image of the MC228-PDMS after stability testing.

Figure 12a illustrates the output voltage of the MW-MC228-TENG during its 600 s operation at different frequencies. Whether it is the human motion frequency ranging from 1 to 3 Hz or the fixed operating frequency of 6 Hz in this experiment, the TENG demonstrates pronounced stability. Figure 12b displays the scanning electron microscopy (SEM) image of the PDMS specimen after the stability test. Following an extended period of operation, the surface microstructure has undergone minimal changes, which suggests that the fabricated specimen possesses good durability.

### 3.6. Force Sensitive and Flexoelectric Effect

**Figure 13:**
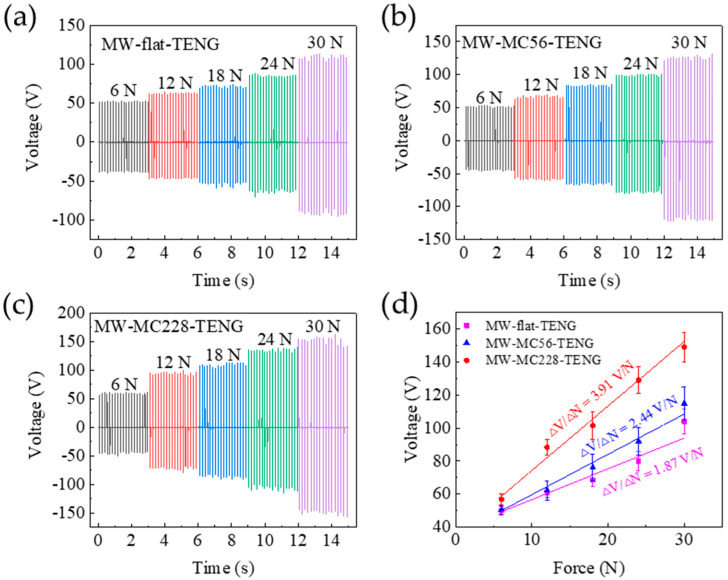
Open circuit voltages for the devices for different applied force: (**a**) MW-flat-TENG, (**b**) MW-MC56-TENG, (**c**) MW-MC228-TENG. The comparison of (**d**) the output voltages and force sensitivity of the three types of TENGs.

In vertical contact–separation mode, applied mechanical stress induces cyclic deformation and elastic recovery of the PDMS microcones in the MW-MC-TENG, modulating the interfacial contact area between triboelectric layers and consequently determining the device’s electrical output characteristics. The output voltage response of all three TENGs to applied forces (6–30 N) was systematically characterized using a pushing platform, as shown in Figure 13a–c. A positive correlation was observed between the applied force (F) and open-circuit voltage, with voltage amplitude increasing monotonically across the tested force range. As the applied force rises, the contact area between the materials and the contact force between the surfaces grow, leading to greater friction and the accumulation of more electrostatic charges between the two surfaces, which in turn enhances the electrical output response of the TENG [42].

Figure 13d presents the voltage comparison with error bars for all three TENG configurations, clearly demonstrating enhanced triboelectric charging in MW-MC-TENG devices with taller microcones. This performance improvement is attributed to the increased contact area and cone deformation achieved under identical applied forces. Systematic characterization of force sensitivity (Figure 13d) demonstrates a 109.1% enhancement from the MW-flat-TENG (1.87 V/N) to the MW-MC228-TENG (3.91 V/N) across the 6~30 N operational range, highlighting the significant performance gains achievable through microcone integration. This result indicates that the force sensitivity of the TENGs increases with the height of the microcones. The planar polymer structure in the MW-flat-TENG results in an output voltage primarily determined by triboelectric effects during operation [38]. Thus, Figure 13a serves as the reference for comparison. Figure 13a,c,d collectively demonstrate the enhanced triboelectric charging in the MW-MC228-TENG. Under varying external pressures, the MW-MC228-TENG exhibits a significantly higher output voltage than the MW-flat-TENG. As the pressure increases, the average voltage difference between the two devices rises from 6.8 V to 45.2 V, indicating the contribution of concurrent flexoelectricity in the MW-MC228-TENG structure [31,43]. As the microcone height increases, their deformation and strain gradient also increase when the two friction surfaces make contact. The polarization intensity resulting from the flexoelectric effect continues to grow, enhancing the sensor’s apparent triboelectric output and sensitivity.

Compared to the TENG force/pressure sensors in Table 3, the MW-MC-TENG exhibits significantly higher sensitivity than conventional TENGs. The observed sensitivity enhancement directly correlates with the MW-MC-TENG’s hierarchical architecture: high-density microcone arrays provide numerous charge trapping sites, bilateral patterning doubles the active triboelectric interfaces, and increased cone height (228 μm vs. 56 μm) geometrically amplifies the deformable contact area, synergistically enhancing electrostatic induction during cyclic operation. The results demonstrate that the proposed MW-MC-TENG in our study can serve as a self-powered energy source for daily applications and provide a sufficient signal response under external forces.

### 3.7. Impact Warning System Combined with MW-MC-TENG

**Figure 14:**
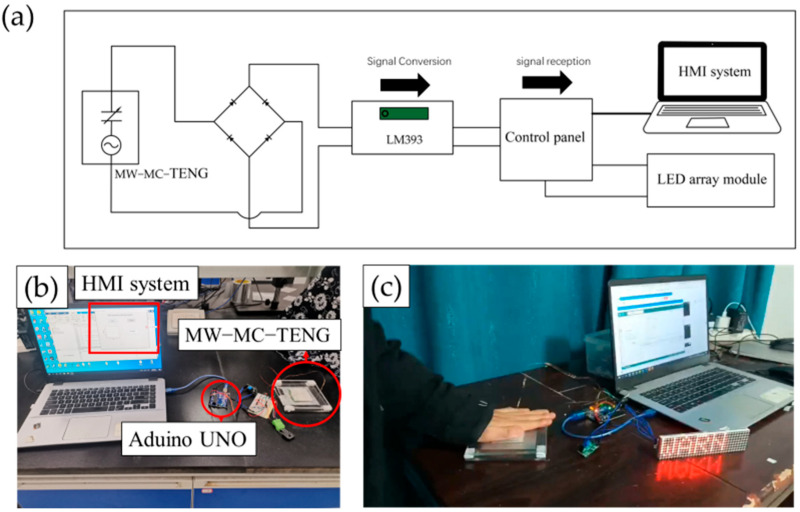
The MW-MC-TENG is used as a self-powered sensor for impacting alarm systems. (**a**) The circuit schematic diagram of the system: The MW-MC-TENG converts the impacting signal into an electrical voltage signal and inputs it into the voltage comparison module, which then generates a signal to the control panel, so that it controls the HMI voice alarm system and visual warning LEDs. After touching, the MW-MC-TENG will output signal to the Arduino UNO. Then, (**b**) the HMI system will send a voice alert and (**c**) the LED array will generate a visual warning of ’Warn’.

As previously mentioned, the MW-MC-TENG can convert mechanical energy into an electrical signal under external stimulation. When connected to a control panel and computing system, it can trigger a response system to relay information to humans when the TENG is activated, such as by touch or impact. In this experiment, an impact warning system is constructed by integrating the MW-MC-TENG, rectifier bridge, LM393 voltage comparison module, Arduino UNO, LED array, and HMI software (V1.0) system created with Matlab 2020, as shown in Figure 14a. We designed two circuit integration approaches: (1) connecting the MW-MC-TENG to an LM393 voltage comparator via a rectifier bridge and then to an Arduino microcontroller, which communicates with a voice human–machine interface (HMI) system on a computer through a serial port; (2) connecting the MW-MC-TENG to an LM393 through a rectifier bridge and then to an Arduino, which controls an LED array screen via a wired connection. The MW-MC-TENG operates as a self-sufficient sensor, transducing mechanical impacts (e.g., from vulnerable individuals) into electrical signals without external power. Signal processing occurs via an LM393 comparator (0.5 V threshold), with exceeded voltages generating a 1.5–2 V output to the Arduino UNO. This triggers multimodal HMI alerts: (1) voice warnings for hazard notification or (2) LED-based visual “WARN” displays (Figure 14b,c, Videos S1 and S2). In this application, the inherent energy generation ability of the MW-MC-TENG eliminates the need for an external power supply, allowing it to function without maintaining a constant powered state. Consequently, compared to traditional sensors, using the MW-MC-TENG as a self-powered sensor offers significant efficiency advantages.

## 4. Conclusions

This study proposes a facile, cost-effective strategy to enhance the electrical output performance of TENGs through dual-sided surface patterning using a CO_2_ laser. The MW-MC-TENG integrates a microcone-patterned PDMS tribo-layer with a microwave-structured Al electrode, fabricated using two PMMA molds with identical micro-hole arrays but varying depths: a deeper mold for PDMS demolding and a shallower one for cold imprinting Al foil. The MW-MC228-TENG demonstrates exceptional electrical output performance, achieving an open-circuit voltage (*V_oc_*) of 157 V, short-circuit current (*I_sc_*) of 78.5 μA, and current density (*J_sc_*) of 3.14 µA/cm^2^ under operational conditions of 6 Hz contact–separation frequency and 20 mm separation distance. This performance outperforms other TENGs, making it the preferred choice for the MW-MC-TENG. The device achieves a peak power density of 16.4 W/m^2^ at an optimal load resistance of 15 MΩ. Furthermore, the MW-MC-TENG demonstrates robust energy harvesting capabilities, successfully charging 0.1 μF and 33 μF capacitors to 2.0 V (1 s) and 1.2 V (45 s), respectively. The device’s practical utility is further evidenced by its ability to simultaneously illuminate 198 commercial LEDs, confirming its effectiveness as an energy source. The MW-MC-TENG demonstrates exceptional force sensitivity (3.91 V/N), establishing its effectiveness as a self-powered force sensor for quantitative pressure monitoring applications. Moreover, it functions as a detection sensor in an HMI impact warning system for intelligent home hazardous area monitoring, helping supervise children or blind individuals by providing visual and voice alerts. The MW-MC-TENG shows significant potential for applications in self-driven sensing and smart home technologies within the IoT ecosystem.

## Figures and Tables

**Table 1:** Comparison of dual-surface modified TENGs.

Morphology	Material	Fabrication Method	Operation Condition	Electrical Characteristics			Ref
				*V_oc_* (V)	*I_sc_* (µA)	*Power Density* (mW/cm^2^)	
Nanoporous-scale morphology	GPA@Nylon -MSF@MXene	Vacuum filtration, etching stripping, and freeze drying	2 kPa, 1 Hz	30	-	-	[25]
Microgroove-microgroove	Cu-PDMS	Oxidation, magnetron sputtering, lithography, and etching	0.5 Mpa, 0.5 Hz	~50	-	-	[26]
Nanofiber-nanofiber	CNT@PDMS-PDMS	Electrospinning, deacetylation, and impregnating	15 N, 10 Hz	94	8.5	0.015	[27]
Sandpapermorphology-nanofiber	ECF-TPUnw	Ecoflex casting	4.4 N 5 Hz	139	4.6	0.16	[28]
Nanoporous-nanoporous	PVDF-UIO@PVDF	Chemistry functionalization, spin coating	9 Hz	-	~50	~0.1225	[29]
Nanopillar-nanopillar	Ni-PDMS	Spin coating, electrodeposition	10 kgf, 3 Hz	~100	~23	-	[30]
Microwave-microcone 180	Al-PDMS	CO_2_ laser ablation and cold imprinting	30 N 6 Hz	141	71.5	1.4	[31]
Microwave-microcone 228	Al-PDMS	CO_2_ laser ablation and cold imprinting	30 N 6 Hz	157	78.5	1.64	ours

**Table 2:** The processing parameters, dimensions, and output performance of three types of TENG.

Combination	Symbol	MW-Flat	MW-MC56	MW-MC228
Patterning Type		Single-Sided	Double-Sided	Double-Sided
Microstructure of Al		microwave (MW)	microwave (MW)	microwave (MW)
Microstructure of PDMS		flat	microcone (MC)	microcone (MC)
Size (cm^2^)		5 × 5	5 × 5	5 × 5
Power of laser (mW)			60	600
Velocity of scanning (mm/s)			11.4	11.4
Ablation pitch (µm)			250	250
Grain density (number of MCs/cm^2^)			1600	1600
Average MC center depth (µm)	H	flat	56	228
Average MC bottom diameter (µm)	D	-	118.6	174.3
Average MC estimated volume (µm^3^)	V	-	205,377.1	1,814,089.9
Average MC estimated surface area (µm^2^)	S	-	15,161.6	66,846.1
Voltage (V)	*V_oc_*	114	127	157
Current (μA)	*I_sc_*	56.5	66	78.5
Current density (µA/cm^2^)	*J_sc_*	2.26	2.64	3.14

**Table 3:** Comparative performance metrics of force and pressure sensors based on TENG technology.

Device	Patterning Type	Triboelectric Material	Fabrication Method	Voltage	Sensitivity	Ref
M-TES	One-side	Latex/FEP-Cu/Acrylic	Multilayer assembly etching	~15 V	0.04 V/kPa	[37]
DP-TENG	One-side	Au/PDMS-Au	ICP etching, Thermal oxidation	~65 V	0.414 V/N	[38]
TENG e-skin sensor	Dual-side	PDMS@AgNWs-PDMS@ PTFE tiny burrs	Replication and molding, spray coating, evaporation and reactive ion etching	3.14 V	0.127 V/kPa	[39]
LU-TEPS	Dual-side	PDMS-PDMS@CNT	Spin coating & curing, evaporation	120 V	0.51 V/kPa	[40]
Two-layer TENG	Dual-side	Al/porous PDMS-Al@Au NPs	Spin coating and curing	40 V	<2.13 V/N	[41]
MW-MC180-TENG	Dual-side	Al-PDMS/Al	CO_2_ laser ablation, cold imprinting	141	3.01 V/N	[31]
MW-MC228-TENG	Dual-side	Al-PDMS/Al	CO_2_ laser ablation, cold imprinting	155 V	3.91 V/N	Ours

## Data Availability

The original contributions presented in the study are included in the article and Supplementary Materials, further inquiries can be directed to the corresponding author.

## Supplementary Materials

The following supporting information can be downloaded at: https://www.mdpi.com/article/10.3390/polym17111569/s1, Video S1: HMI voice alarm; Video S2: LED visual alarm.

## References

[1] Dang V.A., Vu Khanh Q., Nguyen V.H., Nguyen T., Nguyen D.C. (2023). Intelligent Healthcare: Integration of Emerging Technologies and Internet of Things for Humanity. Sensors.

[2] Rehan H. (2023). Internet of Things (IoT) in Smart Cities: Enhancing Urban Living Through Technology. J. Eng. Technol..

[3] Cao X., Xiong Y., Sun J., Xie X., Sun Q., Wang Z.L. (2022). Multidiscipline Applications of Triboelectric Nanogenerators for the Intelligent Era of Internet of Things. Nanomicro Lett..

[4] Parashakti R.D., Perkasa D.H., Aprillita D., Elvaresia M., Rizky M.A. (2024). Human Resource Competency Development to Support the Development of Blue Economy-Based Marine Energy. Ilomata Int. J. Manag..

[5] Shi B., Wang Q., Su H., Li J., Xie B., Wang P., Qiu J., Wu C., Zhang Y., Zhou X. (2023). Progress in recent research on the design and use of triboelectric nanogenerators for harvesting wind energy. Nano Energy.

[6] Pourasl H.H., Barenji R.V., Khojastehnezhad V.M. (2023). Solar energy status in the world: A comprehensive review. Energy Rep..

[7] Rohit R., Kiplangat D.C., Veena R., Jose R., Pradeepkumar A., Kumar K.S. (2023). Tracing the evolution and charting the future of geothermal energy research and development. Renew. Sustain. Energy Rev..

[8] Wang Y., Wang N., Cao X. (2023). From Triboelectric Nanogenerator to Hybrid Energy Harvesters: A Review on the Integration Strategy toward High Efficiency and Multifunctionality. Materials.

[9] Wang Z., Chen Y., Jiang R., Du Y., Shi S., Zhang S., Yan Z., Lin Z., Tan T. (2023). Broadband omnidirectional piezoelectric–electromagnetic hybrid energy harvester for self-charged environmental and biometric sensing from human motion. Nano Energy.

[10] Wang S., Wang C.H., Yuan H.Z., Ji X.P., Yu G.X., Jia X.D. (2023). Size effect of piezoelectric energy harvester for road with high efficiency electrical properties. Appl. Energy.

[11] Chang Y., Huang Y.H., Lin P.S., Hong S.H., Tung S.H., Liu C.L. (2024). Enhanced Electrical Conductivity and Mechanical Properties of Stretchable Thermoelectric Generators Formed by Doped Semiconducting Polymer/Elastomer Blends. ACS Appl. Mater. Interfaces.

[12] Cao R., Wang J., Xing Y., Song W., Li N., Zhao S., Zhang C., Li C. (2018). A self-powered lantern based on a triboelectric–photovoltaic hybrid nanogenerator. Adv. Mater. Technol..

[13] Wang Z.L., Zhu G., Yang Y., Wang S.H., Pan C.F. (2012). Progress in nanogenerators for portable electronics. Mater. Today.

[14] Yang J., Hong K., Hao Y., Zhu X., Qin Y., Su W., Zhang H., Zhang C., Wang Z.L., Li X. (2024). Triboelectric nanogenerators with machine learning for internet of things. Adv. Mater. Technol..

[15] Wen H., Yang X., Huang R., Zheng D., Yuan J., Hong H., Duan J., Zi Y., Tang Q. (2023). Universal Energy Solution for Triboelectric Sensors Toward the 5G Era and Internet of Things. Adv. Sci..

[16] Askari H., Xu N., Barbosa B.H.G., Huang Y., Chen L., Khajepour A., Chen H., Wang Z.L. (2022). Intelligent systems using triboelectric, piezoelectric, and pyroelectric nanogenerators. Mater. Today.

[17] Rawy K., Sharma R., Yoon H.-J., Khan U., Kim S.-W., Kim T.T.-H. (2020). A triboelectric nanogenerator energy harvesting system based on load-aware control for input power from 2.4 μW to 15.6 μW. Nano Energy.

[18] Zhou Y., Shen M., Cui X., Shao Y., Li L., Zhang Y. (2021). Triboelectric nanogenerator based self-powered sensor for artificial intelligence. Nano Energy.

[19] Kim J., Lee D.M., Ryu H., Kim Y.J., Kim H., Yoon H.J., Kang M., Kwak S.S., Kim S.W. (2024). Triboelectric Nanogenerators for Battery-Free Wireless Sensor System Using Multi-Degree of Freedom Vibration. Adv. Mater. Technol..

[20] Yu J., Lv X., Wang K. (2024). Applications of Triboelectric Nanogenerators in Medical Recovery: A Review. ACS Appl. Electron. Mater..

[21] Zhang H., Zhang D., Wang Z., Xi G., Mao R., Ma Y., Wang D., Tang M., Xu Z., Luan H. (2023). Ultrastretchable, Self-Healing Conductive Hydrogel-Based Triboelectric Nanogenerators for Human-Computer Interaction. ACS Appl. Mater. Interfaces.

[22] Si J.H., Duan R.G., Zhang M.L., Liu X.M. (2022). Recent Progress Regarding Materials and Structures of Triboelectric Nanogenerators for AR and VR. Nanomaterials.

[23] Zhou Y.H., Deng W.L., Xu J., Chen J. (2020). Engineering Materials at the Nanoscale for Triboelectric Nanogenerators. Cell Rep. Phys. Sci..

[24] Choi D., Lee Y., Lin Z.H., Cho S., Kim M., Ao C.K., Soh S., Sohn C., Jeong C.K., Lee J. (2023). Recent Advances in Triboelectric Nanogenerators: From Technological Progress to Commercial Applications. ACS Nano.

[25] Zheng C., Gao D.G., Lyu B., Zhang C.G., Li H., Zhou Y.Y., Li N., Ma J.Z. (2023). A triboelectric sensor with highly sensitive and durable: Dual regulation strategy based on surface morphology and functional groups on negative/positive tribolayers. Chem. Eng. J..

[26] Yang W.X., Yang S.Q., Sun Z., Chen P., Qiao X.X. (2024). Regulating the electrical performance of contact-separation mode triboelectric nanogenerators based on double-sided groove textures. J. Micromech. Microeng..

[27] Cao M.Y., Chen Y.L., Sha J., Xu Y.L., Chen S., Xu F. (2024). All-cellulose nanofiber-based sustainable triboelectric nanogenerators for enhanced energy harvesting. Polymers.

[28] Gajula P., Muhammad F.M., Reza M.S., Jaisankar S.N., Kim K.J., Kim H.D. (2023). Fabrication of a silicon elastomer-based self-powered flexible triboelectric sensor for wearable energy harvesting and biomedical applications. ACS Appl. Electron. Mater..

[29] Zhu P.H., Ullah Z., Zheng S.R., Yang Z.R., Yu S.W., Zhu S.P., Liu L.W., He A.H., Wang C.G., Li Q. (2023). Ultrahigh current output from triboelectric nanogenerators based on UIO-66 materials for electrochemical cathodic protection. Nano Energy.

[30] Choi H.J., Lee J.H., Jun J., Kim T.Y., Kim S.W., Lee H. (2016). High-performance triboelectric nanogenerators with artificially well-tailored interlocked interfaces. Nano Energy.

[31] Lin D.Y., Chung C.K. (2024). High-Performance Triboelectric Nanogenerator with Double-Side Patterned Surfaces Prepared by CO_2_ Laser for Human Motion Energy Harvesting. Micromachines.

[32] Ke K.H., Chung C.K. (2020). High-Performance Al/PDMS TENG with Novel Complex Morphology of Two-Height Microneedles Array for High-Sensitivity Force-Sensor and Self-Powered Application. Small.

[33] Ke K.-H., Lin L., Chung C.-K. (2022). Low-cost micro-graphite doped polydimethylsiloxane composite film for enhancement of mechanical-to-electrical energy conversion with aluminum and its application. J. Taiwan Inst. Chem. Eng..

[34] Lin L., Chung C.K. (2021). PDMS Microfabrication and Design for Microfluidics and Sustainable Energy Application: Review. Micromachines.

[35] Chung C., Ke K. (2020). High contact surface area enhanced Al/PDMS triboelectric nanogenerator using novel overlapped microneedle arrays and its application to lighting and self-powered devices. Appl. Surf. Sci..

[36] Chung C.K., Huang Y.J., Wang T.K., Lo Y.L. (2022). Fiber-Based Triboelectric Nanogenerator for Mechanical Energy Harvesting and Its Application to a Human-Machine Interface. Sensors.

[37] Bai P., Zhu G., Jing Q.S., Yang J., Chen J., Su Y.J., Ma J.S., Zhang G., Wang Z.L. (2014). Membrane-Based Self-Powered Triboelectric Sensors for Pressure Change Detection and Its Uses in Security Surveillance and Healthcare Monitoring. Adv. Funct. Mater..

[38] Tcho I.W., Kim W.G., Jeon S.B., Park S.J., Lee B.J., Bae H.K., Kim D., Choi Y.K. (2017). Surface structural analysis of a friction layer for a triboelectric nanogenerator. Nano Energy.

[39] Yao G., Xu L., Cheng X.W., Li Y.Y., Huang X., Guo W., Liu S.Y., Wang Z.L., Wu H. (2020). Bioinspired Triboelectric Nanogenerators as Self-Powered Electronic Skin for Robotic Tactile Sensing. Adv. Funct. Mater..

[40] Rasel M.S., Maharjan P., Salauddin M., Rahman M.T., Cho H.O., Kim J.W., Park J.Y. (2018). An impedance tunable and highly efficient triboelectric nanogenerator for large-scale, ultra-sensitive pressure sensing applications. Nano Energy.

[41] Chun J.S., Ye B.U., Lee J.W., Choi D., Kang C.Y., Kim S.W., Wang Z.L., Baik J.M. (2016). Boosted output performance of triboelectric nanogenerator via electric double layer effect. Nat. Commun..

[42] Min G.B., Xu Y., Cochran P., Gadegaard N., Mulvihill D.M., Dahiya R. (2021). Origin of the contact force-dependent response of triboelectric nanogenerators. Nano Energy.

[43] Qiao H.M., Zhao P., Kwon O., Sohn A., Zhuo F.P., Lee D.M., Sun C., Seol D., Lee D., Kim S.W. (2021). Mixed Triboelectric and Flexoelectric Charge Transfer at the Nanoscale. Adv. Sci..

